# The Interaction of RecA With Both CheA and CheW Is Required for Chemotaxis

**DOI:** 10.3389/fmicb.2020.00583

**Published:** 2020-04-07

**Authors:** Elisabet Frutos-Grilo, Maria Marsal, Oihane Irazoki, Jordi Barbé, Susana Campoy

**Affiliations:** ^1^Departament de Genètica i de Microbiologia, Universitat Autònoma de Barcelona, Barcelona, Spain; ^2^ICFO-Institut de Ciències Fotòniques, The Barcelona Institute of Science and Technology, Barcelona, Spain

**Keywords:** SOS response system, chemotaxis, RecA, CheA, chemoreceptor polar arrays, STED microscopy, swarming

## Abstract

*Salmonella enterica* is the most frequently reported cause of foodborne illness. As in other microorganisms, chemotaxis affords key physiological benefits, including enhanced access to growth substrates, but also plays an important role in infection and disease. Chemoreceptor signaling core complexes, consisting of CheA, CheW and methyl-accepting chemotaxis proteins (MCPs), modulate the switching of bacterial flagella rotation that drives cell motility. These complexes, through the formation of heterohexameric rings composed of CheA and CheW, form large clusters at the cell poles. RecA plays a key role in polar cluster formation, impairing the assembly when the SOS response is activated. In this study, we determined that RecA protein interacts with both CheW and CheA. The binding of these proteins to RecA is needed for wild-type polar cluster formation. *In silico* models showed that one RecA molecule, attached to one signaling unit, fits within a CheA-CheW ring without interfering with the complex formation or array assembly. Activation of the SOS response is followed by an increase in RecA, which rises up the number of signaling complexes associated with this protein. This suggests the presence of allosteric inhibition in the CheA-CheW interaction and thus of heterohexameric ring formation, impairing the array assembly. STED imaging demonstrated that all core unit components (CheA, CheW, and MPCs) have the same subcellular location as RecA. Activation of the SOS response promotes the RecA distribution along the cell instead of being at the cell poles. CheA- and CheW- RecA interactions are also crucial for chemotaxis, which is maintained when the SOS response is induced and the signaling units are dispersed. Our results provide new molecular-level insights into the function of RecA in chemoreceptor clustering and chemotaxis determining that the impaired chemoreceptor clustering not only inhibits swarming but also modulates chemotaxis in SOS-induced cells, thereby modifying bacterial motility in the presence of DNA-damaging compounds, such as antibiotics.

## Introduction

Chemotaxis allows bacteria to sense their environment and adjust their flagellar rotation accordingly, resulting in their directed movement toward attractants and away from repellents (Falke et al., 1997; Bi and Lai, 2015). Chemoreceptors are methyl-accepting chemotaxis proteins (MCPs) that detect the presence of chemoeffectors and modulate the activity of the CheA kinase that, via the CheY chemotaxis response regulator, initiates the signaling pathway controlling the flagellar motor (Sourjik and Wingreen, 2012). There are different types of MCPs, being Tar and Tsr the most abundant and studied in *Salmonella enterica* and *Escherichia coli* (Blat and Eisenbach, 1995). In many *Bacteria* and *Archaea*, MCPs group together to form large chemosensory arrays that contain from a few to thousands of chemoreceptor core complexes (Briegel et al., 2009, 2012, 2015; Greenfield et al., 2009). These ordered structures act as “antennae,” amplifying chemoeffector sensing by cooperative networking (Li and Hazelbauer, 2014; Frank et al., 2016; Piñas et al., 2016). Besides chemotaxis, chemoreceptor clusters are essential for swarming motility (Cardozo et al., 2010; Santos et al., 2014; Irazoki et al., 2016b) and are involved in other important processes, including biofilm formation (He and Bauer, 2014; Huang et al., 2019b), cell adhesion (Huang et al., 2017), host colonization (Erhardt, 2016; Johnson and Ottemann, 2018) and antibiotic resistance (Butler et al., 2010; Irazoki et al., 2017).

In *E. coli* and *S. enterica*, the signaling core complexes are formed by two heterotrimers of transmembrane MCP homodimers, each one coupled to a protomer of the CheA kinase by the chemoreceptor adaptor protein CheW (Li and Hazelbauer, 2004, 2011; Koler et al., 2018). CheA is a dimeric histidine kinase that presents five structural and functional domains associated with: histidine-containing phosphotransfer (P1), CheY/CheB binding (P2), dimerization (P3), ATP binding/catalysis (P4), and CheW binding (P5) (Bilwes et al., 1999). The architecture of the chemoreceptor array has been previously elucidated, in which the interaction between CheW and the P5-CheA domain was shown to be the key structural link between core signaling units in the arrays (Liu et al., 2012, Li et al., 2013; Briegel et al., 2014b; Cassidy et al., 2015; Piñas et al., 2016). Specifically, the interaction of CheW with P5-CheA links three core complexes [using (CheW-CheA_2_-CheW) core linkers] and forms a hexagonal ring of receptors, giving rise to a lattice of hexagonally packed receptor trimers of dimers networked by P5-CheA/CheW rings (Briegel et al., 2012, 2014a; Liu et al., 2012; Cassidy et al., 2015). These highly stable structures are located at the cell poles (Maddock and Shapiro, 1993; Sourjik and Berg, 2000; Jones and Armitage, 2015; Koler et al., 2018).

Several conditions can disrupt chemoreceptor array assembly. In *S. enterica* and *E. coli*, the absence of RecA (Gómez-Gómez et al., 2007; Mayola et al., 2014), the stoichiometric excess of CheW (Cardozo et al., 2010; Irazoki et al., 2016b) and an increase in the concentration of the RecA protein prompted by the activation of SOS response (Irazoki et al., 2016b) inhibit polar chemoreceptor array formation and suppress swarming motility. RecA is a multifunctional protein, it is the main bacterial recombinase and is also involved in DNA repair (Cox, 1999; Lusetti and Cox, 2002; Patel et al., 2010; Keyamura et al., 2013) being the SOS response activator (Little and Mount, 1982; Maslowska et al., 2019). When DNA damage occurs, RecA acquires co-protease activity and thus the ability to promote the auto-cleavage, among others, of LexA, the SOS system repressor. The LexA auto-hydrolysis induces the expression of SOS genes (including *recA*), most of which are involved in DNA repair (Sassanfar and Roberts, 1990). RecA is also essential for chemoreceptor polar array formation and standard flagellar rotation switching (Mayola et al., 2014). In previous work, we showed that RecA interacts with CheW (Irazoki et al., 2016a) impairing chemoreceptor clustering and consequently swarming motility during activation of the SOS response (Irazoki et al., 2016a, b). Specifically, when the SOS response is induced, the intracellular locations of CheW and RecA changes from the poles to along the cell axis (Irazoki et al., 2016a). Only after repair of the DNA damage are the polar arrays restored (Irazoki et al., 2016a, b). However, whether the inhibition of array assembly is due to CheW titration by RecA, thereby altering the stoichiometric balance of these proteins, or to other causes is unclear.

Previous studies showed that the structures of the P5-CheA domain and CheW are paralogous (Vu et al., 2012; Piñas et al., 2018). The mutual substitution of the P5-CheA domain and CheW within the hexagonal rings of chemoreceptors has also been described (Bilwes et al., 1999; Park et al., 2006). Based on these observations, we hypothesized that RecA also interacts with CheA and is part of the chemoreceptor core complexes comprising the chemoreceptor arrays.

Thus, to better understand the association of the SOS response and RecA with chemoreceptor cluster formation and chemotaxis, we explored the interaction between RecA and the P5-CheA domain and identified the region involved in that interaction. In addition, we determined the location within SOS-response-activated cells of the major chemoreceptor core unit-components and the impact of this intracellular distribution on chemotaxis.

## Materials and Methods

### Bacterial Strains and Growth Conditions

Except when indicated, all strains were grown at 37°C in Luria-Bertani (LB) broth or on LB plates, supplemented, when necessary, with ampicillin (100 μg/mL), chloramphenicol (34 μg/mL), and/or kanamycin (10 μg/mL). The strains and constructions used in this work are described in Supplementary Table S1.

### *In silico* Docking Analysis

RaptorX (Källberg et al., 2012) custom-generated *S. enterica* RecA, CheW, Tar, and CheA protein structures were used in the docking assays. In all cases, the available resolved structures of *E. coli* RecA (PDB: 2REB) (Story et al., 1992) and CheW (PDB: 2HO9), and *Thermotoga maritima* Tar and CheA (PDB: 3JA6.C) (Cassidy et al., 2015) were used to validate the obtained 3D structures. *In silico* models were generated using the ClusPro server (Comeau et al., 2004).

The interaction of CheA and RecA was assessed in a simple protein-protein docking study. At least 30 of the highest-scoring models, in which RecA was the receptor and CheA the ligand, and *vice versa*, were analyzed in duplicate. The protein structures and the obtained *in silico* models were visualized and analyzed using PyMOL software (Schrödinger, 2010).

For *in silico* studies of the signaling core unit and ring complex formation, the RaptorX generated structures were compared with the structures documented in *T. maritima* (PDB:3JA6) (Cassidy et al., 2015) and modeled using PyMOL (Schrödinger, 2010), with the RecA protein placed according to the identified residues of the CheW-RecA and CheA-RecA interfaces (Tables 1, 2).

**TABLE 1:** *In vitro* interaction of CheA mutant derivatives with wild-type RecA.

**CheA protein mutated residue^a^**	**CheA domain containing the mutation^b^**	**Interaction with wild-type RecA^c^**
Wild-type	NA^c^	+
M303A	P3	+
L311A	P3	+
G537A	P5, subdomain 1	−
D587A	P5, Subdomain 2	+
K590A	P5, Subdomain 2	−
T591A	P5, Subdomain 2	−
S628A	P5, Subdomain 1	−
S646A	P5, Subdomain 1, Strand β9	−

*NA, not applicable. ^*a*^The mutated residue and the substitution of each tagged mutant derivative are indicated. ^*b*^Unless otherwise indicated, the residue is located in a non-resolved secondary structure region of the P5 CheA domain. ^*c*^Results of co-immunoprecipitation assays using each CheA derivative and wild-type RecA. (+) and (−) indicate the maintenance or abolishment of CheA-RecA complex formation, respectively.*

**TABLE 2:** *In vitro* interaction of RecA mutant derivatives with wild-type CheA and CheW.

**RecA protein^a^**	**Secondary structure region containing the mutated residue**	**Interaction with wild-type CheA^b^**	**Interaction with wild-type CheW^c,d^**
Wild-type	NA	+	+
L10A	Helix α1	+	+
L14A		+	+
Q20A		+	−
H163A	NR	+	+
Q173A	Helix α12	+	+
R176A		+	−
F203	NR	+	+
N213A	Helix α13	+	+
A214V		−	+
K216A		+	+
Y218A		+	+
R222A	Strand β11	−	−
D224A		−	+
I228A		−	+
R243A		+	+
V247A		−	+
K250A		−	−
F255A	NR	+	+
Q257A	Strand β 12	+	+
K286A	NR	+	+
Q300	Strand β 15	+	+

*NA, not applicable; NR, non-resolved secondary structure. ^*a*^The mutated residue and the substitution of each tagged mutant derivative are indicated. ^*b*,*c*^Results of co-immunoprecipitation (CoIP) assays using each RecA derivative and either CheA or CheW wild-type proteins. The maintenance (+) or abolishment (−) of CheA-RecA or CheW-RecA complex formation is shown. ^*d*^Based on the results of previously described CoIP assays (Irazoki et al., 2016a).*

### Construction of RecA and CheA Tagged Proteins and Overexpressing Vectors

Co-immunoprecipitation (CoIP) assays were performed using proteins carrying -6xHis and -FLAG tags. Likewise, -CLIP and -SNAP tagged proteins were used for STED microscopy. Plasmids harboring the corresponding tagged genes were constructed using the appropriate oligonucleotides (Supplementary Table S2) and the HiFi DNA assembly cloning kit (NEB). The tag sequences were included at the 3′ end of the genes preceded by a 3 × Gly linker (Supplementary Figure S1). All PCR products were digested, cloned into the pUA1108 overexpression vector (Mayola et al., 2014) and transformed into *E. coli* DH5α. The *recA* and *cheA* tagged mutants were obtained using a site directed mutagenesis kit (Agilent). All constructions were confirmed by PCR and sequencing. In all cases, the expression of the corresponding tagged derivative was confirmed by Western blotting (Supplementary Figure S2).

### Co-immunoprecipitation Assays

The CoIP assays were conducted as described previously (Mayola et al., 2014; Irazoki et al., 2016a) with a few modifications. Briefly, cultures of *S*. *enterica* Δ*recA*Δ*cheA* carrying the corresponding overexpression plasmid encoding a *recA*, or *cheA* tagged gene and their corresponding mutant derivatives were used (Supplementary Table S1). *S. enterica* Δ*recA*Δ*cheW* background was used when *cheW* tagged gene was induced. In all cases, the tagged-gene overexpression was induced by the addition of 1mM of IPTG and cell lysates were obtained by sonication (Branson Digital Sonifier). As control, cells lysates harboring the pUA1108 overexpression vector were obtained following the same procedure.

The CoIP assays were performed using Pure Proteome Protein A magnetic beads (Millipore) coated, following the manufacturer’s instructions, with either mouse anti-FLAG IgG or anti-6xHis IgG monoclonal primary antibodies (Sigma-Aldrich). Before CoIP, the coated magnetic beads were pre-incubated with the control lysates to minimize non-specific interactions. Two cell lysates containing the corresponding proteins were mixed and incubated at 30°C for 1 h without shaking to allow protein-protein interaction and kept at 4°C without shaking to maintain specific interactions for 16 h. Afterward, treated coated magnetic beads were added to the lysate mixture for 1 h at RT with gently shaking. Magnetic beats were then recovered, washed three times and heated for 10 min at 90°C. Supernatants were separated by SDS-PAGE on a 15% polyacrylamide gel and analyzed by Western blotting using mouse anti-6xHis IgG1 (Merck) and rabbit anti-FLAG^®^ (Merck) and horseradish-peroxidase (HRP)-coupled anti-mouse IgG or anti-rabbit IgG antibodies (Acris). The membranes were developed using a HRP chemoluminiscent substrate (SuperSignalTM West Pico PLUS Chemiluminescent Substrate, Thermo Scientific) following the manufacturer’s instructions. The membranes were imaged using a ChemiDocTM XRS + system (Bio-Rad).

### Construction of *S. enterica* Mutant and Tagged Strains

*S. enterica* Δ*cheA* and *S. enterica* Δ*cheA*Δ*cheW* mutants and -SNAP and/or -CLIP tagged strains were constructed according to the λRed recombinase-based gene replacement method (Datsenko and Wanner, 2000; Supplementary Figure S1). pGEM-T vectors (Promega) containing -SNAP or -CLIP tags from pSNAP-tag (T7)-2 and pCLIPf vectors (NEB) followed by the kanamycin cassette from pKD4 vector (Datsenko and Wanner, 2000) were constructed using HiFi DNA assembly cloning kit (NEB) giving rise to pUA1135 and pUA1136, respectively (Supplementary Table S1). These plasmids were used as the template to amplify the -SNAP or -CLIP tag followed by the kanamycin cassette using the suitable oligonucleotides (Supplementary Table S2). The PCR products were transformed into the corresponding *S. enterica* cells containing the pKOBEGA plasmid (Chaveroche et al., 2000). When necessary, the antibiotic resistance cassettes were eliminated using the pCP20 plasmid (Datsenko and Wanner, 2000).

The *S. enterica* Δ*recA*Δ*cheA* and Δ*recA*Δ*cheA*Δ*cheW* strains were constructed by transduction as previously described (Campoy et al., 2002), using the P22int7(HT) bacteriophage and *S. enterica* Δ*recA* (UA1927), as donor strain (Mayola et al., 2014). The absence of the prophage in the transductants was determined by streaking them onto green plates as described previously (Davis et al., 1980).

In all cases, gene substation in all constructs was verified by PCR using suitable primers followed by sequencing.

### Residue Conservation Percentage

To determine the percent conservation of the involved residues, all complete genomes from *Salmonella* specie and one random genome of each genus of the *Enterobacteriaceae* family were downloaded from the GenBank database. RecA, CheA, and CheW *S. enterica* ATCC 14028 protein sequences (ACY89831.1, ACY88793.1, and ACY88792.1, respectively) were used as queries in the Basic Local Alignment Search Tool (tBLASTn) to identify similar protein sequences, limited using the previously searched genomes. RecA matches were obtained for 501 *Salmonella* and 23 *Enterobacteriaceae* genomes (Supplementary Data Sheet S1), CheA matches for 492 *Salmonella* and 13 *Enterobacteriaceae* genomes (Supplementary Data Sheet S2) and CheW matches for 501 *Salmonella* and 13 *Enterobacteriaceae* genomes (Supplementary Data Sheet S3). The results were filtered based on cut-offs for the *e*-value (<10^–20^) and coverage (>75%). Multiple sequence alignment and data analysis were carried out using the Clustal Omega local server with standard parameters (Sievers et al., 2011). The data were represented in a heat map obtained using Prism (GraphPad).

### Stimulated Emission Depletion (STED) Microscopy

*S. enterica* cells were labeled using SNAP-Cell^®^ 505-Star and CLIP-Cell^TM^ TMR-Star permeable dyes, which specifically recognize SNAP- and CLIP- tags, respectively, following the manufacturer’s instructions. All strains were cultured and coverslip mounted as described (Irazoki et al., 2016a), supplemented when required with 0.08 μg mitomycin C/mL. Previous STED imaging, samples were examined under an AxioImager M2 microscope (Carl Zeiss Microscopy) to ensure that at least 90% of the cells were correctly labeled.

Fluorescence immunolabeling was carried out as described (Buddelmeijer et al., 2013), with a few modifications. The two-color labeled samples were observed using a commercial gated-STED microscope (Leica TCS SP8 STED 3X) equipped with a pulsed white light laser source and three depletion lasers. STED illumination of cells tagged with SNAP-Cell^®^ 505-Star was performed using a 505 nm line, and depletion using a 592 nm line. For the CLIP-Cell^TM^ TMR-Star tag, the illumination line was 555 nm and the depletion source 660 nm. Fluorescent light was collected using high-efficiency single-molecule detectors (SMD-HyD), using a HC PL APO CS2 100×/1.40 oil objective. The selected areas were scanned at 600 Hz and the final pixel size was 20 nm.

The selected cells were screened along the *z* axis and the brightest plane was chosen. At least three different representative cells were obtained for each sample. The images were deconvoluted using the Lightning GPU-based Deconvolution Leica package. Images for publication were processed and prepared using Fiji ImageJ software (National Institutes of Health).

### Chemotaxis Capillary Assays

Chemotaxis assays were conducted as previously described (Mayola et al., 2014). Briefly, 1 μL capillary tubes (Microcaps, Drummond Scientific Co.) filled with either tethering buffer or 10 mM L-aspartate dissolved in tethering buffer (Block et al., 1983) were placed in contact with 2 mL of the corresponding cell suspension in the chemotaxis chambers formed by placing three V-shaped bent needles (40 mm 18G needle, Nipro). After incubation at 30°C for 1 h, the exterior of capillaries was rinsed under a stream of sterile distelled water. Then capillary tubes were emptied and the cell concentration was determined by plating. Chemotaxis ratios were calculated as the ratio of viable bacteria inside capillary tubes with vs. without aspartate.

### Chemoreceptor Polar Clustering Assay

The chemoreceptor polar cluster arrays were visualized as previously described (Mayola et al., 2014), with a few modifications. Briefly, overnight cultures of the corresponded tagged strains were grown at 30°C in tryptone broth, supplemented, when needed, with ampicillin and/or 40 μM IPTG. Overnight cultures were diluted 1:100 in tryptone broth supplemented with IPTG and incubated at 30°C until an OD_600_ of 0.08–0.1 was reached, Then cells were collected by centrifugation, washed once using ice-cold tethering buffer (10 mM potassium-phosphate pH 7, 67 mM NaCl, 10 mM Na-lactate, 0.1 mM EDTA, and 0.001 mM l-methionine) and resuspended in 20–100 μL of the same buffer. Then cells were stained with the permeable dyes SNAP-Cell^®^ 505-Star and CLIP-Cell^TM^ TMR-Star, following the manufacturer’s instruction. Finally, cells were fixed with paraformaldehyde, resuspended in 1 × PBS, mounted on 35 mm poly-L-lysine-pre-coated coverslips using Mowiol-DABCO mounting medium and air-dried.

The samples were examined under an Axio Imager M2 microscope (Carl Zeiss Microscopy) equipped with the appropriate filter set [green channel: GFP (Zeiss filter set 38); red channel: Rhod (Zeiss filter set 20)]. Cell fields were photographed and at least 350 cells were visually inspected. All images were acquired under identical conditions. Each experiment was performed at least in triplicate using independent cultures; a minimum of 1,050 cells from each studied strain were therefore analyzed. The images presented in the figures are representative of the entire image set. ImageJ software (National Institutes of Health) was used to quantify the number of clusters and to prepare images for publication.

### Statistical Analysis

The results of the chemotaxis and chemoreceptor-clustering assays were statistically evaluated using a one-way analysis of variance (ANOVA) with Prism (GraphPad), as previously described (Brennan et al., 2013; Raterman and Welch, 2013; Mayola et al., 2014). The analyses were followed by the Bonferroni multiple comparison *post hoc* test. A *p*-value < 0.01 was considered to indicate statistical significance. In all cases, the error bars in the figures indicate the standard deviation.

## Results

### RecA and CheA Interaction

**FIGURE 1:**
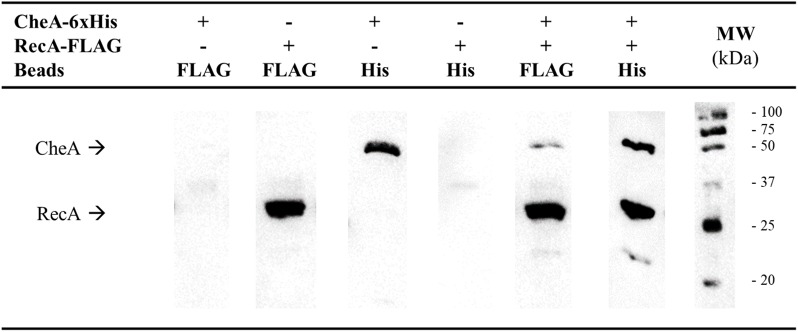
Co-immunoprecipitation assays of *S. enterica* RecA and CheA. Cell-lysates prepared from *S. enterica* Δ*recA*Δ*cheA* cultures overexpressing either RecA-FLAG- or CheA-6xHis-tagged proteins were incubated together to allow interaction of the proteins. Co-immunoprecipitation (CoIP) was performed by adding magnetic beads coated with anti-FLAG (Beads FLAG) or anti-6xHis antibodies (Beads His), attached proteins were recovered and separated by SDS-PAGE. The CoIP controls consisted of mixtures containing only RecA-FLAG or CheA-6xHis overexpressing lysates. The presence in the recovered supernatants of each tagged-protein was assessed by Western blotting using both anti-FLAG and anti-6xHis primary antibodies. The presence or absence of RecA-FLAG, CheA-6xHis, or both tagged proteins in the corresponding lysate is indicated. The experiments were done at least in triplicate. Black arrows show the position of CheA-6xHis and RecA-FLAG. +, added protein; -, non-added protein; MW, molecular mass marker, in kDa.

Given the structural similarities of the P5-CheA domain and CheW (Vu et al., 2012; Piñas et al., 2018), we conducted co-immunoprecipitation (CoIP) assays to determine whether, as with CheW, RecA is able to interact with CheA (Arifuzzaman et al., 2006; Mayola et al., 2014). Thus, RecA-FLAG and CheA-6xHis tagged proteins were overexpressed in *S. enterica* Δ*recA*Δ*cheA* strains carrying the corresponding plasmids (Supplementary Table S1). When both recombinant proteins were present in the protein mixture, anti-FLAG antibody-coated beads recovered both RecA-FLAG and CheA-6xHis from the supernatants (Figure 1). When anti-6xHis antibody-coated beads were added to the mixture, RecA-FLAG proteins were also recovered along with CheA. These results demonstrated the *in vitro* pairing of RecA and CheA.

**FIGURE 2:**
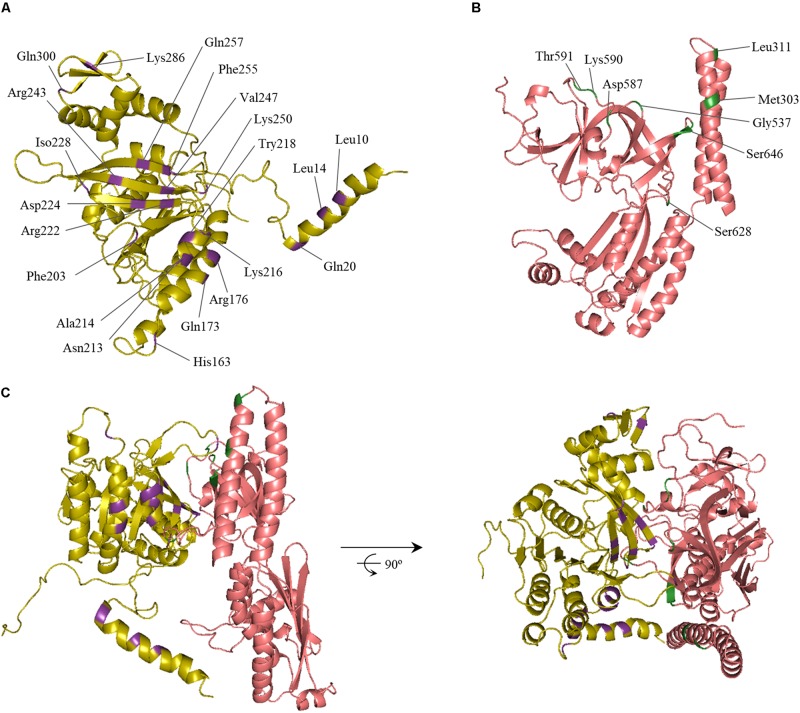
*In silico* model of the RecA and CheA protein interaction. The predicted ternary structures of **(A)** *S. enterica* RecA (yellow) and **(B)** CheA (P3-, P4-, and P5-domains, pink) proteins are shown. The predicted interface of RecA and CheA is highlighted in purple and green, respectively. Residues selected for site-directed mutagenesis and their locations are also indicated. **(C)** Ribbon diagrams of one of the highest-scoring models of the RecA-CheA interaction. The two views of the interaction, obtained using Pymol software, are rotated 90° about the x-axis.

An *in silico* modeling experiment was then conducted, aimed at identifying the putative RecA and CheA residues participating in the interaction of these proteins. Protein-protein interaction docking was performed with RaptorX (Källberg et al., 2012) using, as reference structures, the *E. coli* RecA (PDB: 2REB) (Story et al., 1992) and the *T. maritima* CheA (PDB: 3JA6.C) (Cassidy et al., 2015), which includes the P3-, P4-, and P5-CheA domains. Balanced-coefficient docking models were considered to be the most accurate for the analysis of the RecA-CheA interaction (Comeau et al., 2004). Thirty of the highest-scoring models were analyzed for each combination of RecA receptor protein and CheA ligase and for the reverse combination. Although the spatial arrangement was not exactly the same in each combination, the putative interacting regions were considered to be those repeated in all of the studied models (Figure 2).

As expected, the results were similar to those obtained for the CheW-RecA interaction. In CheA, both P5 subdomains (1 and 2) interacted with RecA. In some of the *in silico* models, residues of the P3 domain were also exposed to the RecA-CheA interface (Figure 2 and Table 1). With respect to RecA, the putative interface with CheA was located in the NH_2_-terminal and central domains (at α1, α12, α13, β11, and β15) (Figure 2 and Table 2).

**FIGURE 3:**
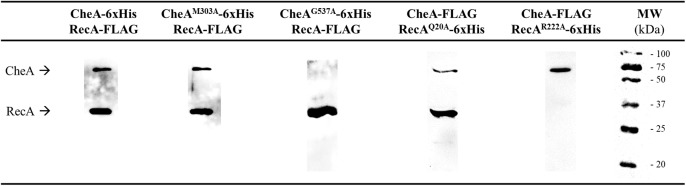
Co-immunoprecipitation assays of *S. enterica* RecA and CheA mutant derivatives. Representative images of the CoIP of mutant derivatives that allow (CheA M303A or RecA R20A) or impair (CheA G537A or RecA R222A) RecA-CheA interaction. Each lane contains a mixture of *S. enterica* Δ*recA*Δ*cheA* cell lysates containing the corresponding 6xHis-tagged overexpressed mutant derivative and the wild-type FLAG-tagged protein. The immunoprecipitates were obtained using anti-FLAG coated magnetic beads. All experiments were done at least in triplicate. The results obtained with all mutant derivatives (Tables 1 and 2) were the same as those shown in the figure. Black arrows indicate RecA and CheA protein bands. NA, not added; MW, molecular mass marker, in kDa.

To confirm these interaction interfaces, site-directed mutagenesis was used to construct the corresponding mutant derivatives for each protein. The 21 RecA and 8 CheA residues were selected based on their exposure and their potential ability to mediate RecA-CheA pair formation (Tables 1, 2). With the exception of the RecA A214V mutant, in which the Ala residue was changed to a Val, all selected residues were converted to an Ala (Tables 1, 2), which is considered to be non-reactive amino acid (Cunningham and Wells, 1989). The corresponding *recA* and *cheA* gene mutants constructed *in vitro* were 6xHis-tagged and the effects of the substitutions on the RecA-CheA interaction were determined by CoIP assays using the corresponding FLAG-tagged wild-type RecA or CheA protein (Figure 3). The results are summarized in Tables 1 and 2. For CheA, only P5 domain was associated with the RecA interaction; CheA mutations in the P3 domain did not disturb wild-type RecA binding (Table 1). Within the P5-CheA domain, five residues (G537, K590, T591, S628, and S646) were found to be directly involved in the interaction with RecA. Their substitution by Ala prevented RecA-CheA pair formation (Table 1 and Figure 3). Analyses of the 21 RecA mutants showed that only five were unable to bind wild-type CheA (A214V, R222A, D224A, K250A, and I228A). With the exception of A214, all of the residues were located on the β11 strand, shown in previous studies to be associated with monomer-monomer interactions as well as RecA filament formation and stabilization (Skiba et al., 1999; Zaitsev and Kowalczykowski, 1999; Chen et al., 2008). Recombinase assays with the RecA mutants showed, in almost all cases, a clear decrease in the recombination activity of the residues associated with RecA-CheA pair formation (Supplementary Figure S3).

However, not all of the residues involved in the CheW-RecA interaction were also associated with the CheA-RecA interaction. Thus, RecA Q20A and R176A mutants, while unable to bind CheW (Irazoki et al., 2016a), interacted with wild-type CheA (Table 2 and Figure 3). Similarly, the involvement of residues A214, D224, and I228 was limited to the RecA-CheA interaction, as they had no effect on RecA-CheW binding (Table 2). Only two mutant derivatives, R222A and K250A, abolished the interactions of CheA and CheW with RecA (Table 2). These results not only revealed the residues associated with RecA-CheA pairing but also demonstrated the ability of RecA to interact with both CheA and CheW through different interfaces. In addition, when CheA and CheW proteins were not present RecA protein was majorly not located at the cell poles (Supplementary Figure S4).

**FIGURE 4:**
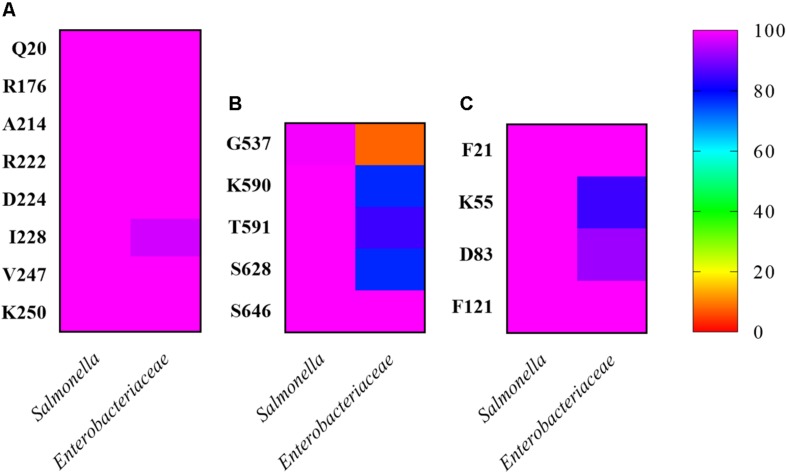
Conservation of RecA-associated residues among *Salmonella* and other representative *Enterobacteraceae*. The percentage of conserved residues in the interaction between **(A)** RecA, **(B)** CheA, and **(C)** CheW from *S. enterica* was calculated based on the number of residues in the studied sequences that differed from the query sequence. The studied residues were compared with their homologs in *Salmonella* and in one representative of each available genus within the family *Enterobacteriaceae*. Identities closer to 0% are shown in red, and those closer to 100% in pink. Intermediate percentages are represented by other colors in the legend. All sequences were downloaded on November 14, 2019.

In addition, we determined the residue conservation percentages among *Salmonella* and *Enterobacteriaceae* for each involved amino acid (Figure 4). The RecA residues involved in CheA and CheW interactions were highly conserved (100% identity; Figure 4A), except for residue I228 (96.4%). Among the CheA residues associated with the RecA interaction, all were conserved in *Salmonella* (100% identity), except G537, which differed in *S. bongori*, resulting in a slightly lower identity (99.2%). The CheA residues were also highly conserved in *Enterobacteriaceae* (>75% identity), again except G537 (7.7%). Finally, for the involved residues of CheW, the results were similar, with 100% identity in *Salmonella* and >80% in *Enterobacteriaceae*. According to these findings, the ability of RecA to interact with CheW and CheA may occurs not only in *Salmonella* species besides *S. enterica* but also in *Enterobacteriaceae*. These results pointed out that the association of the SOS response with chemoreceptor signaling complexes may be extended to *Enterobacteriaceae* and perhaps also to other families of bacteria.

### RecA as a Part of the Chemoreceptor Signaling Core Unit

**FIGURE 5:**
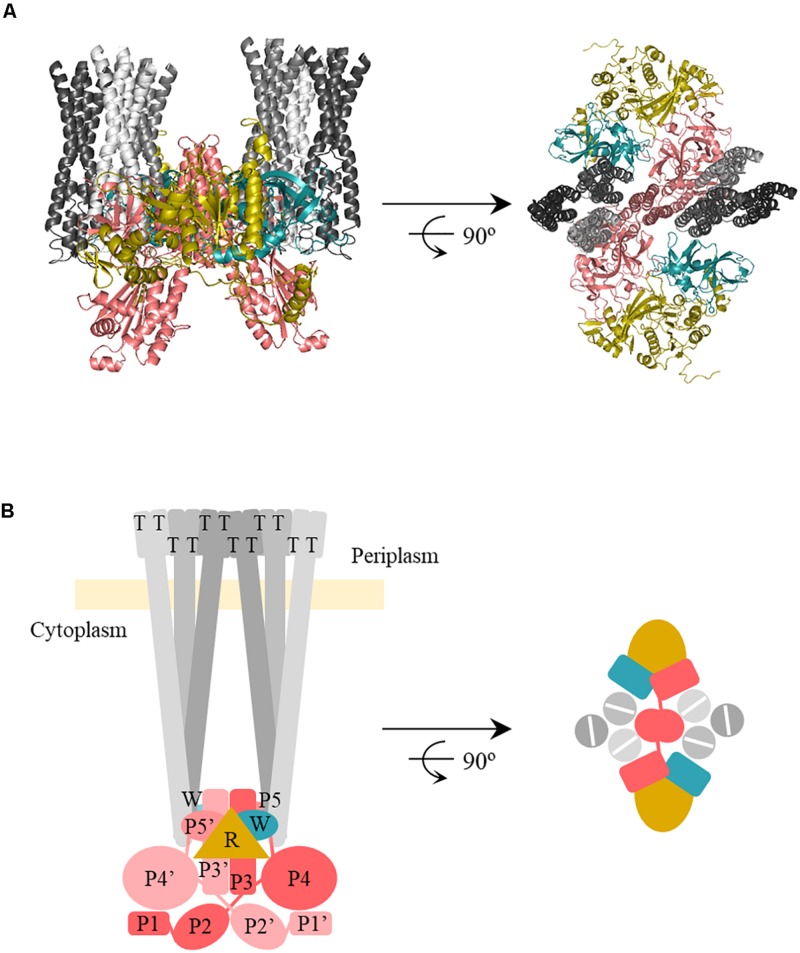
*In silico* model of the interaction of RecA-CheA-CheW proteins forming the core signaling complex. **(A)** The predicted ternary structures of *S. enterica* RecA (R, yellow), CheW (W, blue), CheA (P3-P5, pink), and Tar (T, gray) are represented in cartoon form. Model images are cross-sections through the receptor tip and CheA/CheW baseplate, viewed perpendicular (left) and parallell to (right) the cytoplasmatic membrane. The proteins were modeled using Pymol software according to the studied protein-protein interfaces responsible for RecA-CheW (Irazoki et al., 2016a), RecA-CheA (in this study), and CheA-CheW-Tar (Piñas et al., 2016) interactions. **(B)** Schematic representation of the above *in silico* model and also including the P1 and P2 domains.

Our results also indicated the differential interaction of RecA with CheW and CheA (Table 2). RecA interfaces with CheA and CheW do not overlap with the regions of CheA-CheW binding, nor with those involved in MCP interaction (Cassidy et al., 2015; Piñas et al., 2016; Huang et al., 2019a). These observation suggested that RecA may be part of a signaling complex, a possibility explored by generating *in silico* interaction models that included the entire signaling core unit (Figure 5). The RaptorX-generated structures for all *S. enterica* proteins were compared with the structure of the *T. maritima* chemotaxis signaling complex (PDB:3JA6) (Cassidy et al., 2015) and modeled using PyMOL software (Schrödinger, 2010). The RecA interaction was placed according to the residues determined to be directly involved in the CheA-RecA or CheW-RecA interfaces (Tables 1, 2). As seen in Figure 5, the RecA fits into the chemoreceptor signaling complex without allosteric interference.

### RecA Interacts With Both CheW and CheA *in vivo*

To corroborate the results of the *in silico* models and study the importance of the RecA-CheA interaction for chemoreceptor polar cluster formation *in vivo*, the location of CheA and Tar proteins was analyzed using the stimulated emission depletion microscopy (STED), a super-resolution fluorescence imaging technique that increases the axial resolution of biological samples up to 20–40 nm (Han and Ha, 2015).

*S. enterica cheA:SNAP tar:CLIP* and Δ*recA cheA:SNAP tar:CLIP* tagged strains were constructed and were tested in chemoreceptor clustering and swarming assays under non-DNA damage conditions to verify that tag addition did not alter their chemoreceptor array phenotypes. No changes in either chemoreceptor polar clusters or swarming motility (Cardozo et al., 2010; Partridge et al., 2019) were observed for *S. enterica cheA:SNAP tar:CLIP* strain. Its phenotype was the same as that of *S. enterica* wild-type (Supplementary Figure S5). Also, *S. enterica* Δ*recA cheA:SNAP tar:CLIP* was unable to swarm and the number of chemoreceptor polar clusters was drastically reduced (Supplementary Figure S5) since RecA is essential for both swarming and polar array cluster formation (Mayola et al., 2014).

The RecA complementation and overexpression assays were performed using the pUA1108 vector containing wild-type *recA* and its derivatives unable to interact with CheA (RecA A214V) or CheW (RecA R222A) or both proteins (RecA R176A) under the control of the Ptac IPTG-inducible promoter. The plasmids were transformed into *S. enterica cheA:SNAP tar:CLIP* and Δ*recA cheA:SNAP tar:CLIP* strains (Supplementary Table S1), and the intracellular location of CheA and Tar was then determined.

The SNAP and CLIP tags are self-labeling enzymes derived from the human DNA repair protein *O*^6^-alkylguanine-DNA alkyltransferase. Appropiate permeable dyes directly attach to the target protein with high reactivity and labeling specificity. The SNAP-tag binds *O*^6^-benzylguanine derivatives (Keppler et al., 2003), and the CLIP-tag *O*^2^-benzylcytosine derivatives (Gautier et al., 2008). Due to these differences, the two tags, with their permeable dyes suitable for STED imaging (SNAP-Cell^®^ 505-Star and CLIP-Cell^TM^ TMR-Star, respectively), can be employed simultaneously to specifically label target proteins in living cells (Gautier et al., 2008).

**FIGURE 6:**
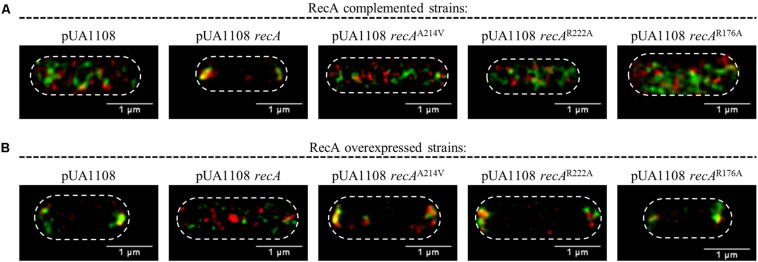
Representative STED images of the locations of CheA and Tar within *S. enterica*. Cells containing the pUA1108 expression vector, either empty or carrying wild-type *recA*, a non-CheA interacting *recA* mutant derivative (A214V), a non-CheW interacting (R222A) or a *recA* mutant derivative that interacts with neither CheA nor CheW (R176A) are shown. The corresponding plasmids were included in the genetic backgrounds of **(A)** *S. enterica* Δ*recA cheA:SNAP tar:CLIP* and **(B)** *S. enterica cheA:SNAP tar:CLIP*. CheA and Tar proteins were labeled with the permeable dyes CLIP-Cell^TM^ TMR-Star and SNAP-Cell^®^ 505-Star, respectively. For all images, overlapped channel results are presented. The maximum intensity projection images of the obtained *z*-stacks are shown. All experiments were done at least in triplicate.

The absence of RecA impairs chemoreceptor array formation (Mayola et al., 2014). For complementation assays, the basal expression of the wild-type *recA* gene cloned in the pUA118 vector was enough to restore chemoreceptor array formation and cell CheA and Tar were located again in cell poles (Figure 6A; Mayola et al., 2014). Nevertheless, in the presence of a non-CheA-interacting RecA, no chemoreceptor polar clusters were formed (Figure 6A) and CheA and Tar were distributed along the cell. The same phenotype was observed using a RecA mutant unable to interact with CheW (Figure 6A) or with both CheA and CheW (Figure 6A).

On the other hand, the RecA overexpression inhibited polar cluster assembly in a wild-type genetic background (Irazoki et al., 2016b). Then, as expected, the IPTG-induced expression of a wild-type *recA* gene within *S. enterica cheA*:*SNAP tar:CLIP* promoted the redistribution of CheA and Tar along the cell (Figure 6B). However, the increased expression mediated by IPTG of *recA* mutants unable to bind CheA, CheW or both proteins did not alter the CheA and Tar location, that remained at the cell poles (Figure 6B). Together, the results indicate that the interaction of RecA with both CheA and CheW is needed for chemoreceptor polar array formation and that both interactions occur *in vivo*.

### Location of CheA and Tar Proteins Within SOS Response-Induced Cells

In bacteria grown in liquid medium under non-DNA damaging conditions, the polar cluster array proteins CheW, CheA, and Tar are located mainly at the cell poles (Sourjik and Berg, 2000; Greenfield et al., 2009; Koler et al., 2018). Several studies have shown that RecA also localizes at the poles (Lusetti and Cox, 2002; Lesterlin et al., 2014; Irazoki et al., 2016b). Our previous work demonstrated that during SOS response activation, RecA and CheW are no longer located at the cell poles but in small foci distributed along the cell (Irazoki et al., 2016a). To further understand the association of RecA with CheA and the signaling core units, the location of CheA and Tar proteins in SOS-response-induced cells was studied in *S. enterica cheA:SNAP tar:CLIP* and *cheA:SNAP recA:CLIP* tagged strains by STED imaging.

**FIGURE 7:**
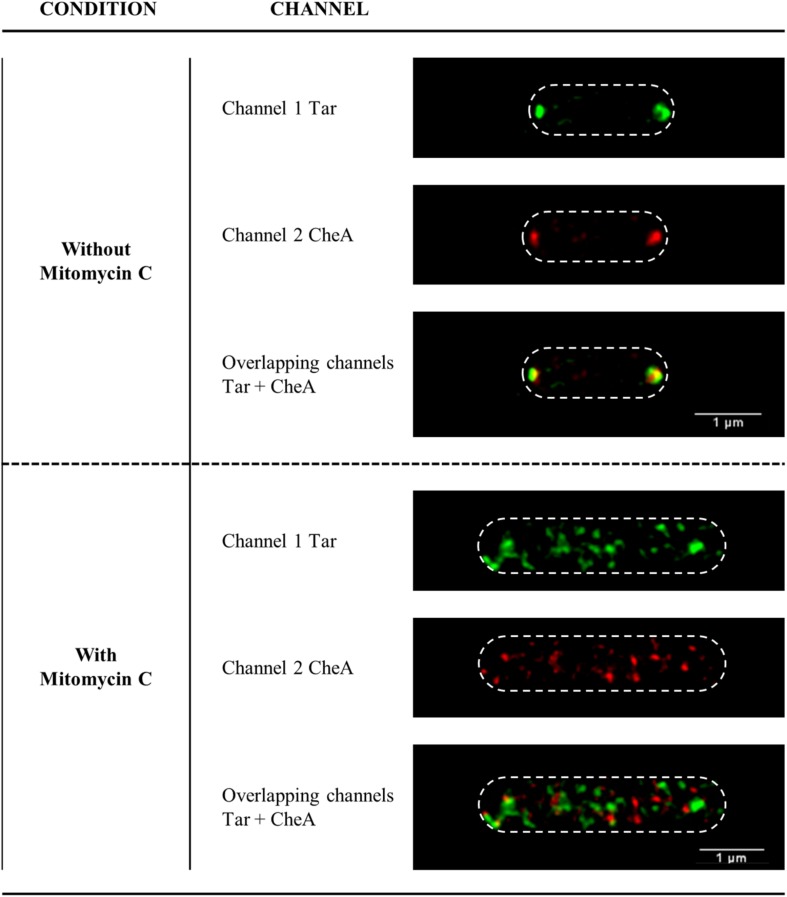
Representative STED images of the subcellular locations of Tar and CheA in *S. enterica cheA:SNAP tar:CLIP* cells in the absence or presence of SOS inducer. The Tar and CheA proteins were labeled with the permeable dyes CLIP-Cell^TM^ TMR-Star (channel 1, in green) and SNAP-Cell^®^ 505-Star (channel 2, in red), respectively. When appropriate, mitomycin C was added at a final concentration of 0.08 μg/mL. For all images, each channel is shown both individually and overlapped. The maximum intensity projection images of the z-stacks are shown. All experiments were done at least in triplicate.

Under non-DNA-damaging condition, RecA, CheA, and Tar were, as expected, located at the poles of *S. enterica* cells (Figure 7 and Supplementary Figure S6). However, the addition of a sublethal concentration of SOS-inducer resulted in cell filamentation and the redistribution of CheA and Tar (Figure 7) to follow that of RecA (Supplementary Figure S6) and CheW (Irazoki et al., 2016a). Thus, under non-DNA-damaging conditions and during activation of the SOS response, the intracellular distributions of CheA, CheW, Tar and RecA were the same.

### SOS Response-Induced Cells Present Normal Chemotaxis Response

To further demonstrate that RecA is a component of the signaling core unit and that the structure of this unit is preserved during SOS response activation, chemoreceptor polar clustering and chemotaxis assays were performed by exposing cells of different genetic backgrounds to a sublethal concentration of mitomycin C and then monitoring the chemotaxis response.

**FIGURE 8:**
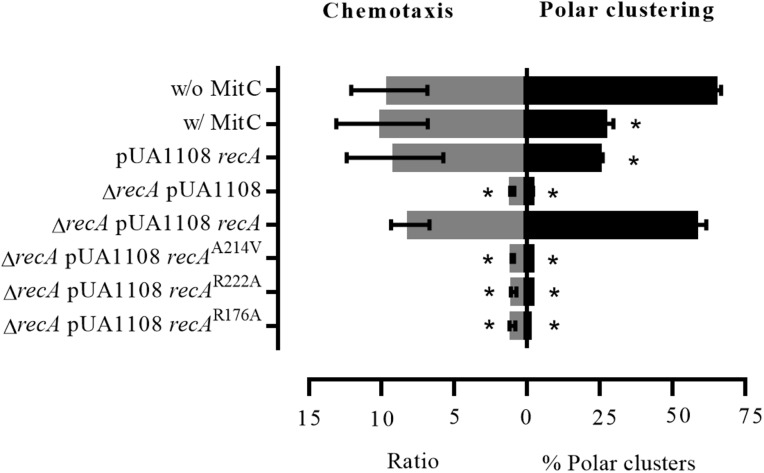
Chemotaxis ability and chemoreceptor polar clustering of *S. enterica* strains. Chemotaxis assay results and the percentage of chemoreceptor polar clusters in *S. enterica cheA:SNAP tar:CLIP* and Δ*recA cheA:SNAP tar:CLIP* strains. As needed, the cells were grown in the absence (w/o MitC) or presence (w/MitC) of mitomycin C (0.08 μg/mL). When indicated, they were transformed with either empty pUA1108 or the plasmid carrying wild-type *recA* or a *recA* mutant derivative [non-CheA-interacting (A214V), non-CheW-interacting (R222A) or interacting with neither CheA nor CheW (R176A)]. The chemotaxis ratios were calculated as the ratio of viable bacteria inside capillary tubes with vs. without aspartate. The results are the mean of three independent experiments. Error bars represent the standard deviation.

As shown in Figure 8, the presence of mitomycin C did not affect *S. enterica* wild-type strain chemotaxis. The same results were obtained when the wild-type *recA* was overexpressed in bacterial cells by the addition of IPTG. Under these conditions, i.e., in the presence of mitomycin C or *recA* overexpression, the absence of chemoreceptor polar clusters did not lead to an inhibition of the chemotaxis response. Interestingly, in the presence of mitomycin C, chemoreceptor polar array formation in the Δ*recA* strain are lower than that observed in either SOS-induced or RecA-overexpressing wild-type cells (Figure 8). Further, as previously published (Mayola et al., 2014), Δ*recA* cells were unable to respond to a chemoeffector, an ability that was restored only by the addition of wild-type *recA*. Neither polar cluster array formation nor chemotaxis (Figures 6, 8) were restored in a Δ*recA* strain complemented with *recA* mutant derivatives unable to interact with CheA, CheW or both. According to these results, the formation of active signaling core units and therefore chemoreceptor polar arrays requires the binding of RecA to both CheA and CheW.

## Discussion

Our results provide strong evidence supporting the interaction of RecA with P5-CheA domain (Figure 1), which is structurally similar to CheW (Vu et al., 2012; Table 1). While both P5-CheA subdomains 1 and 2 participate in the RecA-CheA interaction (Table 1), the involved CheA-residues do not overlap with those of the CheA-CheW interaction (Cassidy et al., 2015). For RecA, the CheA binding interface is located at its NH_2_-terminus, between residues 214 and 250 (Figure 2 and Table 2). This region is mainly associated with monomer-monomer interaction as well as RecA filament formation and stabilization (Skiba et al., 1999; Zaitsev and Kowalczykowski, 1999; Chen et al., 2008). Moreover, almost all of the residues involved in RecA-CheA pair formation had a very low recombinase activity (Supplementary Figure S3).

*In silico* docking established that the RecA interaction could be fitted to the chemoreceptor signaling complex without any allosterical interference (Figure 5), as the P5-CheA subdomain 1 was still able to interact with CheW subdomain 2 (also known as interface 1) (Natale et al., 2013). Despite the similarity of CheW and P5-CheA, only RecA Arg222 and Lys250 residues, located at the β11 strand, were associated with both RecA-CheA and RecA-CheW pair formation (Table 2; Irazoki et al., 2016a). The rest of the identified RecA residues (Ala214, located in the α13 helix, and Asp224, Ile228, and Val247, all of them in the β11 strand) are only associated with CheA binding, and their mutation did not affect the RecA-CheW interaction (Bilwes et al., 1999; Cassidy et al., 2015). RecA binding to CheW is mediated not only by the β11 strand but also by the α1 and α12 helices of RecA (Table 2; Irazoki et al., 2016a). As in the RecA-CheA interaction, RecA-CheW pairing does not interfere with the binding of any of the other CheW binding partners identified so far (CheA, CheW, and MCPs) (Irazoki et al., 2016a). Moreover, all residues involved in RecA-CheA and RecA-CheW interfaces were highly conserved not only in *S. enterica* but also in other members of *Enterobacteriaceae* (Figure 4).

*In vivo* assays showed that chemoreceptor polar clustering requires the interaction of RecA with both CheA and CheW. Indeed, RecA was distributed along the cell when polar clusters are not built (Supplementary Figure S4). Further, RecA mutants unable to bind CheA, CheW, or both proteins neither restored wild-type chemoreceptor polar cluster assembly in cells with a Δ*recA* genetic background (Figure 6A) nor abolished polar cluster formation in wild-type cells overexpressing RecA (Irazoki et al., 2016b; Figura 6B). According to these results, the interaction of RecA not only with CheW but also with CheA is essential for chemoreceptor array formation.

Previous studies showed that the stoichiometry of chemoreceptor core unit components within the cell is crucial for polar array assembly. For example, the absence or overexpression of CheW abolishes chemoreceptor cluster formation (Avram Sanders et al., 1989; Cardozo et al., 2010). The same phenotype occurs in a knock out *recA* mutant (Mayola et al., 2014) or when the RecA concentration is increased, whether by SOS response activation or by its overexpression (Gómez-Gómez et al., 2007; Irazoki et al., 2016b). In earlier work, we proposed that RecA prompts the titration of CheW, thus preventing chemoreceptor assembly and, in turn, polar cluster array formation during activation of the SOS response (Irazoki et al., 2016a). The same sequence of events may describe the CheA-RecA interaction. Nevertheless, the results described herein clearly determine that RecA protein present different binding interfaces with CheA and CheW, that do not overlap with those associated with CheA-CheW interaction or with their binding to MCPs. Together with the fact that, in the absence of RecA there is no chemotaxis response (Mayola et al., 2014), suggested the direct interaction of RecA with the chemoreceptor core unit.

STED imaging of the tagged strains indicated that RecA, CheA, Tar (Figure 6 and Supplementary Figure S6) and CheW (Irazoki et al., 2016a), the main components of the signaling core unit, follow the same intracellular distribution when RecA concentration is increased following the activation of the SOS response or *recA* overexpression, moving from the cell poles to along the cell axis (Figure 6 and Supplementary Figure S6; Irazoki et al., 2016a). Furthermore, the polar cluster arrays of *S. enterica* Δ*recA* cells were not restored by the presence of a *recA* mutant unable to bind CheA, CheW, or both, such that chemotaxis was inhibited (Figure 8). Only the addition of wild-type RecA reestablished chemotaxis (Figure 8). Taken together, these findings demonstrate that RecA and its ability to bind CheA and CheW are crucial for the functionality of the signaling core complex and for chemotaxis.

**FIGURE 9:**
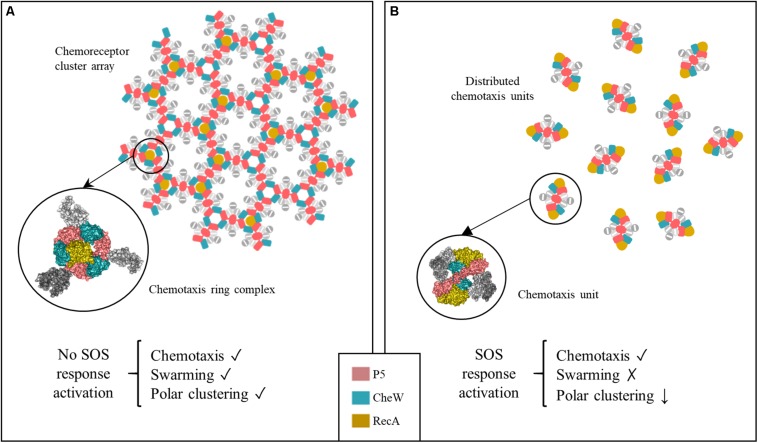
Proposed model of chemoreceptor assembly inhibition during activation of the SOS response. **(A)** Under non-DNA damaging conditions, hexagonally assembled chemoreceptor polar clusters are formed by three signaling core units that are stabilized by P5-CheA-CheW rings. Only one RecA molecule fits within the inner aspect of the heterohexameric ring. Under this condition, the cells are able to swarm, polar cluster arrays are formed and the cell exhibits chemotaxis. **(B)** Activation of the SOS response is followed by a high increase in the RecA concentration. This induces an increase in the number of RecA-associated signaling complexes. In that context, the heterohexameric ring formation is allosterically disturbed by the presence of more than one RecA molecule. As a result, chemoreceptor polar clusters are unable to form and chemoreceptor signaling units remain distributed along the cell. These cells are unable to swarm but retain a chemotaxis response.

The direct association of RecA with the chemoreceptor core unit also explains the impaired formation of chemoreceptor polar clusters in the presence of increased RecA (Figure 9). Chemoreceptor arrays consist of hexagonal lattices of MCPs stabilized by interconnected heterohexameric rings of CheA and CheW (Cassidy et al., 2015). The rings are formed by the alternated interactions between P5-CheA and CheW of three unit core complexes that give rise to interface 2, composed of the P5-CheA subdomain 2 and CheW subdomain 1. This key link between signaling core complexes in the array is needed for cluster assembly (Natale et al., 2013; Cassidy et al., 2015). As shown in Figure 9, there is enough space within a ring to fit one RecA molecule interacting with one of the three internal face of (CheW-CheA_2_-CheW) heterohexameric ring, without altering its hexagonally structure. When SOS response activation increases the intracellular RecA concentration, the protein becomes associated with a greater number of signaling core units. However, the build-up of heterohexameric rings is prevented by the high levels of RecA, which impair the formation of CheW-P5-CheA interface 2 and consequently, the array assembly is inhibited.

It is important to note that the chemotaxis response is not associated with the presence of polar chemoreceptor clusters, unlike swarming. Thus, in the *S. enterica* wild-type strain, while either SOS response activation by mitomycin C or the overexpression of wild-type *recA* impaired chemoreceptor array formation and therefore swarming motility (Supplementary Figure S7; Irazoki et al., 2016b), there was no effect on chemotaxis (Figure 8). The signaling core units were completely functional, even in the absence of chemoreceptors arrays, only in the presence of wild-type RecA, not a RecA mutant unable to bind CheA, CheW, or both (Figure 8). Our results indicates, in agreement with previously reported data, that although the absence of polar clustering clearly impairs swarming ability (Cardozo et al., 2010; Frank et al., 2016; Irazoki et al., 2016b), the chemotaxis pathway remains functional (Maki et al., 2000; Briegel et al., 2014b; Frank et al., 2016; Piñas et al., 2016). These results are in Further, it is also known that chemotaxis is not affected when *E. coli* cells are treated with cephalexin (Maki et al., 2000), a β-lactam antibiotic that induces the SOS response (Bano et al., 2014).

The chemotaxis of bacterial cells is enhanced by the networking of chemoreceptors (Frank et al., 2016). Nevertheless, Frank et al. (2016) showed that the CheA kinase levels and flagellar motor switching are similar in cells with and without polar clusters. The same authors found that, under conditions of high CheA kinase activity, cells with dispersed receptor complexes are more sensitive to chemoeffectors than cells with polar clusters (Frank et al., 2016). However, cells can remodel their chemotaxis signaling pathway to enhance swarming motility (Partridge et al., 2019). The over production of RecA due SOS response activation by the presence of DNA-damaging compounds or antibiotics, increase the number of signaling core units associated to RecA, and consequently modulate the architecture of chemoreceptor arrays, by impairing the heterohexameric CheA-CheW ring formation (Figure 9). Thus, the distribution of chemoreceptor signaling units modulates not only swarming motility in cells growing on a surface, to prevent exposure to higher concentrations of SOS-inducer compounds (Irazoki et al., 2016b), but also the chemotaxis performance of the SOS-induced cells. The high degree of conservation of residues associated with the CheA-RecA-CheW interaction in *Enterobacteriaceae* (Figure 4) suggested that the relationship between the SOS response, chemotaxis and swarming to modulate bacterial motility in the presence of antibiotics and other injurious or potentially lethal compounds may be extended to other species of this bacterial group.

## Data Availability Statement

All datasets generated for this study are included in the article/Supplementary Material.

## Author Contributions

EF-G, OI, and SC performed the *in silico* analyses and site-directed mutagenesis. EF-G and MM performed the fluorescent immunolabeling and STED imaging. EF-G and SC designed, performed, and analyzed the rest of experiments. EF-G, SC, and JB coordinated the research, discussed the findings, and interpreted the results. SC wrote the first draft of the manuscript. EF-G, MM, and OI wrote sections of the manuscript. All authors contributed conception and design of the study, contributed to manuscript revision, read and approved the submitted version.

## Conflict of Interest

The authors declare that the research was conducted in the absence of any commercial or financial relationships that could be construed as a potential conflict of interest.
