# Supplementary material for: The Interaction of RecA With Both CheA and CheW Is Required for Chemotaxis
Source: Front Microbiol. 2020 Apr 7;11:583. doi: 10.3389/fmicb.2020.00583 (PMC7154110; doi:10.3389/fmicb.2020.00583)
Supplement: Supplementary file 1 [file Image_1.pdf]

Supplementary Material

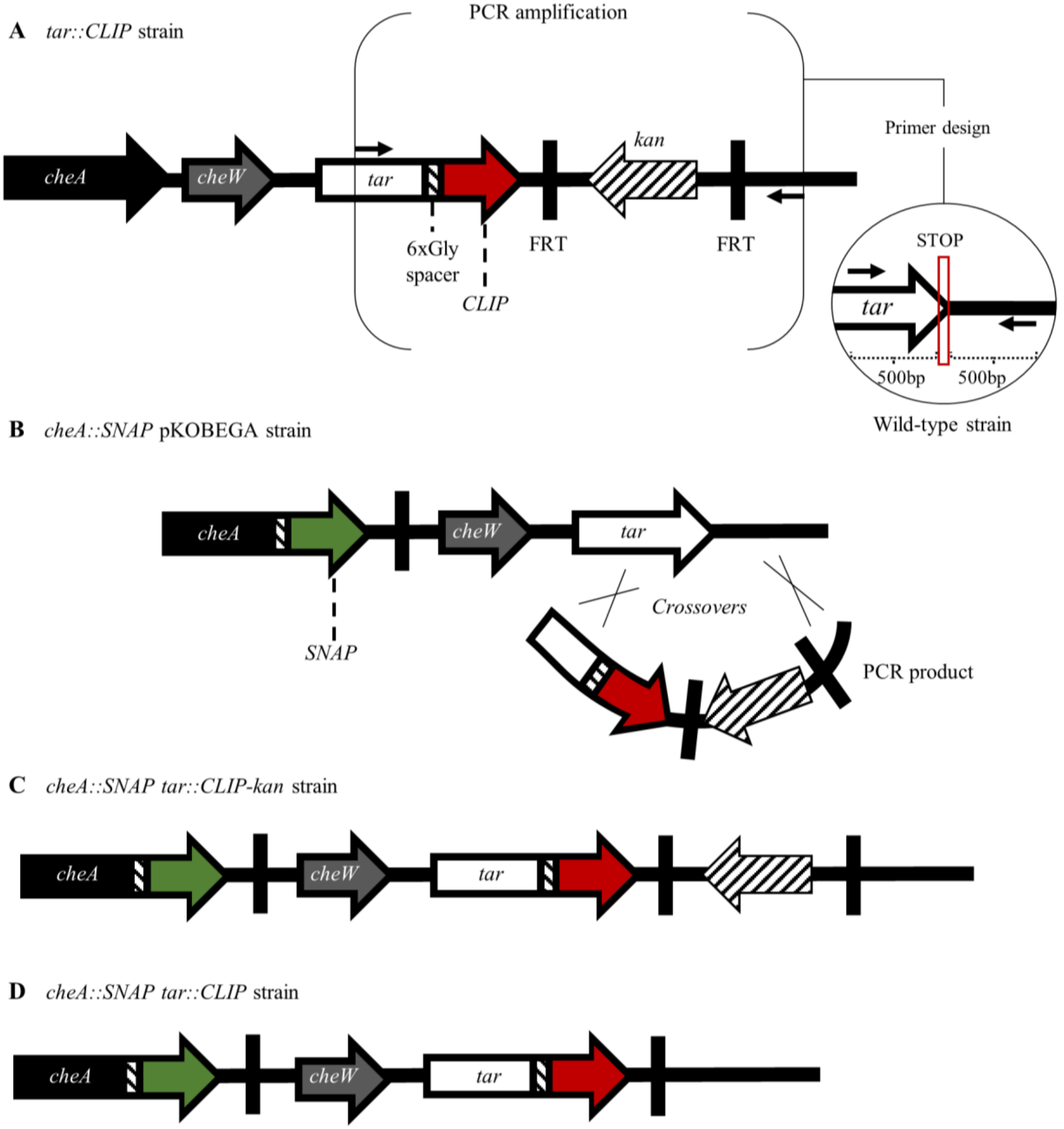

Supplementary Figure 1. Schematic representation of the *S. enterica cheA::SNAP tar::CLIP* strain construct obtained using the λ Red recombinase-based gene replacement method. **(A)** *S. enterica tar::CLIP* was constructed by introducing the tag between the last nucleotide and the STOP triplet of the *tar* gene. To construct the double-tagged strain, a PCR with primers amplifying 500 bp upstream and downstream of the *tar* STOP codon was performed. **(B)** The PCR product was electroporated into the *S. enterica cheA::SNAP* pKOBEGA strain and double recombination allowed the tagging of *tar* with CLIP, generating the strain **(C)** *S. enterica cheA::SNAP tar::CLIP-kan*. **(D)** Kanamycin resistance was removed using pCP20, yielding the *cheA::SNAP tar::CLIP* strain.
