# Supplementary material for: The Interaction of RecA With Both CheA and CheW Is Required for Chemotaxis
Source: Front Microbiol. 2020 Apr 7;11:583. doi: 10.3389/fmicb.2020.00583 (PMC7154110; doi:10.3389/fmicb.2020.00583)
Supplement: Supplementary file 2 [file Image_2.pdf]

Supplementary Material

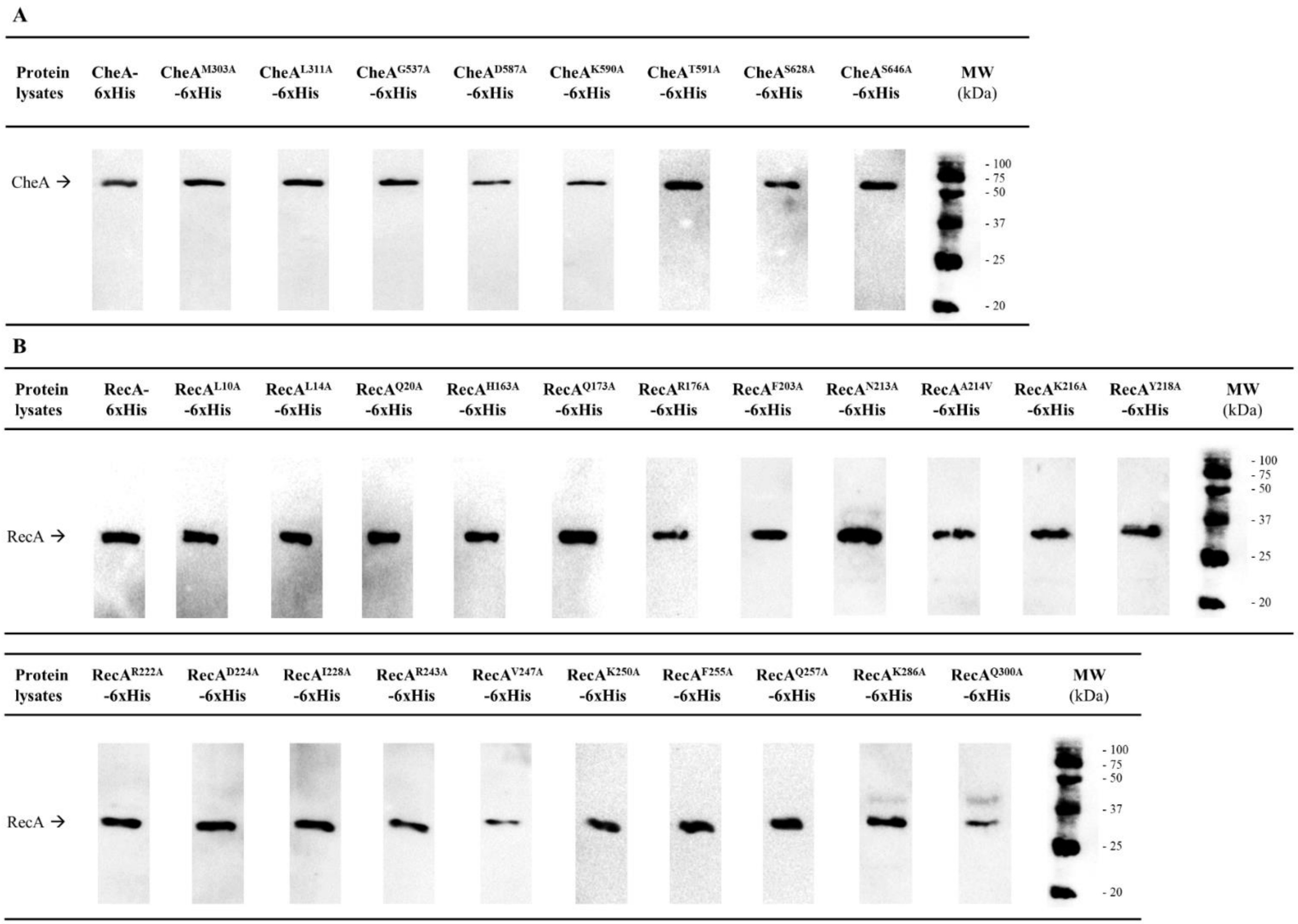

Supplementary Figure 2. Expression of RecA and CheA mutant derivatives. Western blot assays of the cell lysates expressing the corresponding *S. enterica* **(A)** CheA or **(B)** RecA mutant derivative were performed. In both cases, CheA and RecA proteins were detected using anti-6xHis IgG1 (Merck) and horseradish-peroxidase (HRP)-coupled anti-mouse IgG (Acris) was added as secondary antibody.
