# Supplementary material for: The Interaction of RecA With Both CheA and CheW Is Required for Chemotaxis
Source: Front Microbiol. 2020 Apr 7;11:583. doi: 10.3389/fmicb.2020.00583 (PMC7154110; doi:10.3389/fmicb.2020.00583)
Supplement: Supplementary file 3 [file Image_3.pdf]

Supplementary Material

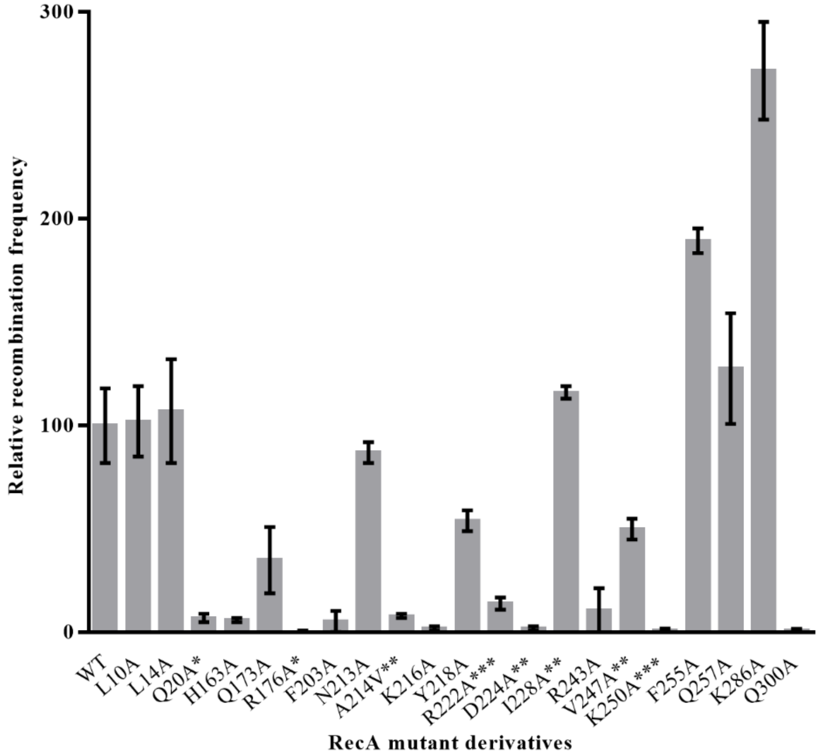

Supplementary Figure 3. ***In vivo* recombination activity of the RecA mutant derivatives**. The efficiency of *S. enterica* Δ*recA* strains containing an expression vector carrying the corresponding *recA* mutant derivative and transduced by bacteriophage P22intH7 was tested for use in recombination studies using a selectable genetic marker. The method was performed as described previously (Irazoki et al., 2016). The relative recombination frequency was calculated as the recombination efficiency of each mutant derivative with respect to that of the strain overexpressing wild-type *recA*. The recombination efficiency of each strain is the number of transductants compared with the initial recipient cell concentration. RecA mutant derivatives unable to interact with CheW, with CheA or both proteins are indicated by asterisks (*, ** or *** respectively). The relative recombination frequencies were calculated as the mean of three independent experiments. Error bars indicate the standard deviations.

Irazoki, O., Aranda, J., Zimmermann, T., Campoy, S., and Barbé, J. (2016). Molecular interaction and cellular location of RecA and CheW proteins in *Salmonella enterica* during SOS response and their implication in swarming. *Front. Microbiol.* 7, 1560. doi:10.3389/fmicb.2016.01560.
