# Supplementary material for: The Interaction of RecA With Both CheA and CheW Is Required for Chemotaxis
Source: Front Microbiol. 2020 Apr 7;11:583. doi: 10.3389/fmicb.2020.00583 (PMC7154110; doi:10.3389/fmicb.2020.00583)
Supplement: Supplementary file 4 [file Image_4.pdf]

Supplementary Material

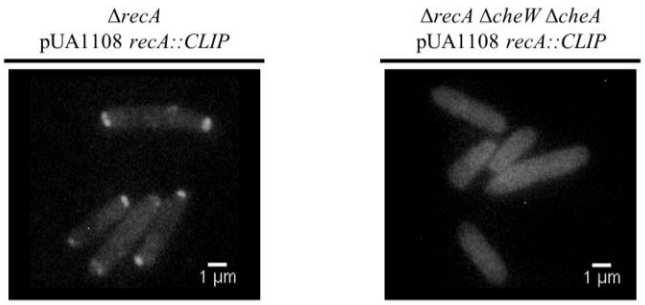

Supplementary Figure 4. RecA localization in the absence of CheA and CheW. Representative fluorescence images of Δ*recA* and Δ*recA* Δ*cheW* Δ*cheA* strains complemented with a pUA1108 plasmid containing a *recA*::*CLIP*. RecA protein was labelled with the permeable dyes CLIP-Cell™ TMR-Star. The samples were examined under an Axio Imager M2 microscope (Carl Zeiss Microscopy) equipped with the Rhod (Zeiss filter set 20) filter set.
