# Supplementary material for: The Interaction of RecA With Both CheA and CheW Is Required for Chemotaxis
Source: Front Microbiol. 2020 Apr 7;11:583. doi: 10.3389/fmicb.2020.00583 (PMC7154110; doi:10.3389/fmicb.2020.00583)
Supplement: Supplementary file 5 [file Image_5.pdf]

frontiers

Supplementary Material

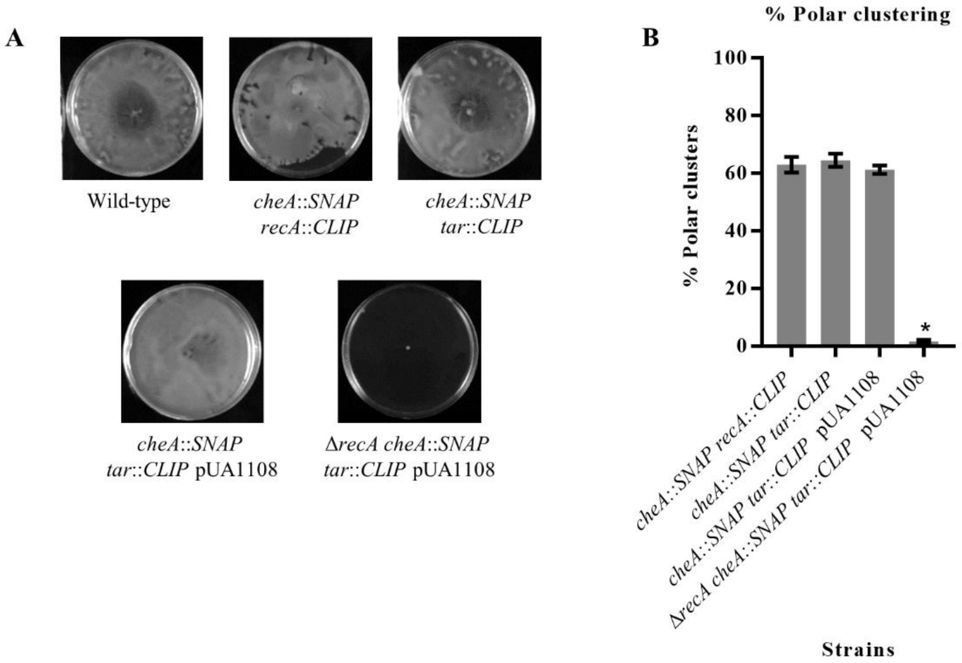

Supplementary Figure 5. Swarming assays and chemoreceptor polar clustering of *S. enterica* tagged strains. (A) Swarming motility and **(B)** chemoreceptor polar clustering were assayed to confirm that addition of the corresponding tag had no effect on the phenotype of the tested *S. enterica* strains. Swarming assays were performed as previously described (Mayola et al., 2014). The experiment was done at least in triplicate. Images of the chemoreceptor clustering assays were acquired under identical conditions and at least 350 cells were visually inspected to determine the presence and types of clusters in each sample. The results are the mean of at least three independent imaging studies. Then, a minimum of 1050 cells from each studied strain were analysed. Error bars represent the standard deviation. *$p<0.01$ as determined in a one-way ANOVA with a Bonferroni correction.

Mayola A, Irazoki O, Martínez IA, Petrov D, Menolascina F, Stocker R, Reyes-Darias JA, Krell T, Barbé J, Campoy S. 2014. RecA protein plays a role in the chemotactic response and chemoreceptor clustering of *Salmonella enterica*. PLoS One 9:e105578.
