# Supplementary material for: The Interaction of RecA With Both CheA and CheW Is Required for Chemotaxis
Source: Front Microbiol. 2020 Apr 7;11:583. doi: 10.3389/fmicb.2020.00583 (PMC7154110; doi:10.3389/fmicb.2020.00583)
Supplement: Supplementary file 6 [file Image_6.pdf]

Supplementary Material

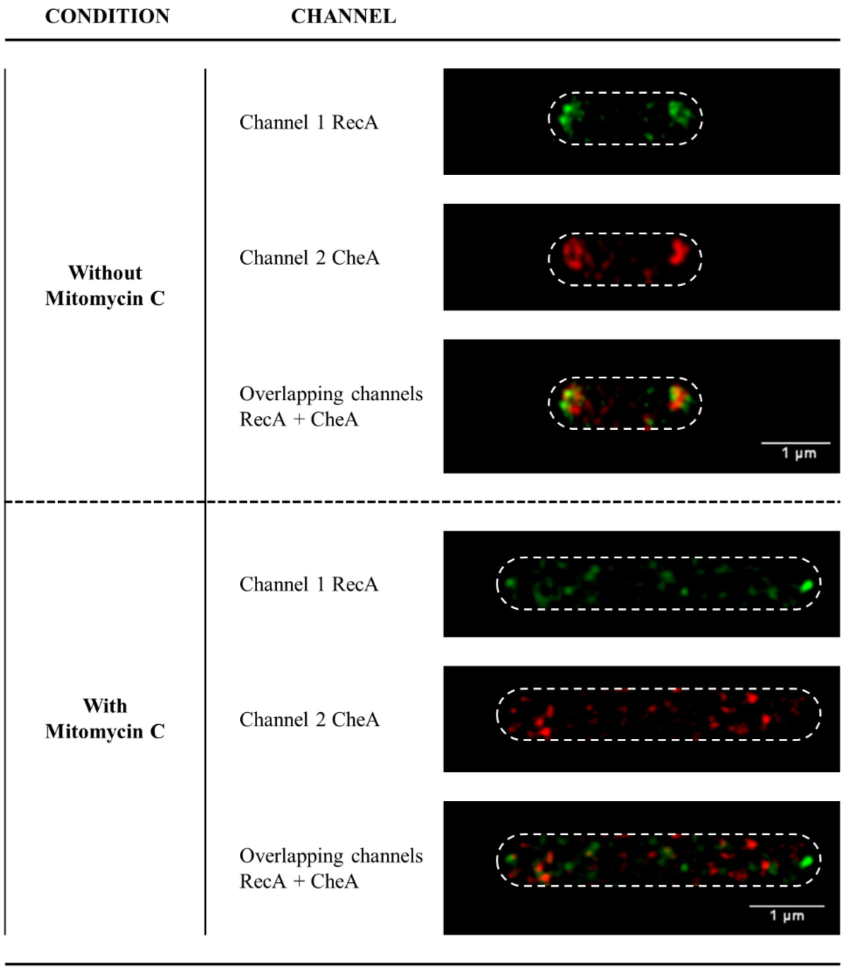

Supplementary Figure 6. Representative STED images of the subcellular locations of CheA and RecA in *S. enterica cheA*::*SNAP recA*::*CLIP* cells in the absence or presence of SOS inducer (0.08 µg mitomycin C/mL). RecA and CheA proteins were labeled with the permeable dyes CLIP-Cell™ TMR-Star (channel 1, represented in green) and SNAP-Cell® 505-Star (channel 2, in red), respectively. For all images, each channel is shown both individually and overlapped. The maximum intensity projection images of the obtained z-stacks are also shown. All experiments were done at least in triplicate.
