# Supplementary material for: The Interaction of RecA With Both CheA and CheW Is Required for Chemotaxis
Source: Front Microbiol. 2020 Apr 7;11:583. doi: 10.3389/fmicb.2020.00583 (PMC7154110; doi:10.3389/fmicb.2020.00583)
Supplement: Supplementary file 7 [file Image_7.pdf]

Supplementary Material

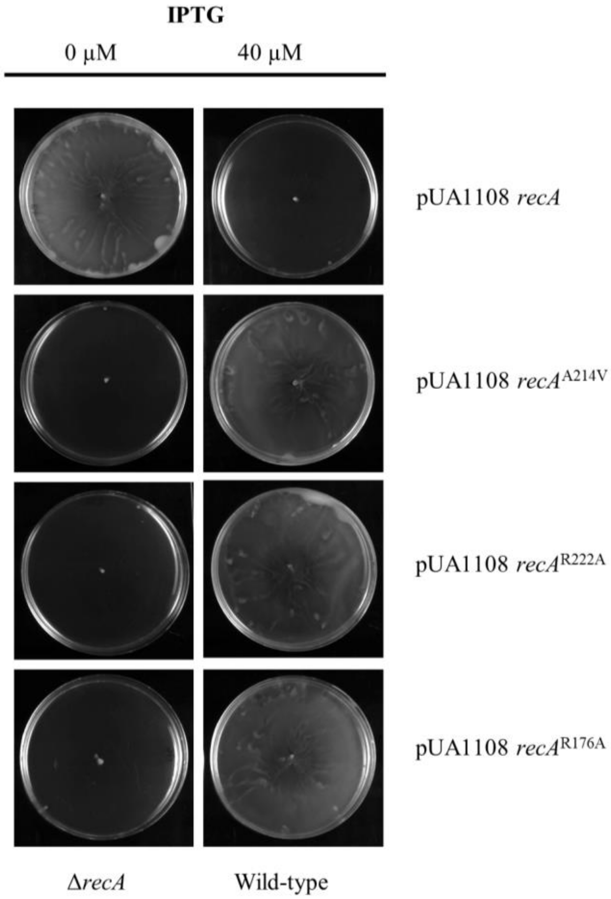

Supplementary Figure 7. Swarming assays from *S. enterica* Δ*recA* and wild-type strains containing pUA1108 carrying the *recA* gene or the corresponding mutant derivative. Cells are able to complement the lack of RecA and restore swarming ability only with the plasmid containing the wild type gene, but not if RecA cannot interact with CheA and/or CheW within the Δ*recA* strain. By contrast, in cells with basal expression of RecA (Wild-type), an increase in cytosolic wild-type RecA abolishes swarming, whereas swarming is maintained in the presence of the other RecA mutant derivatives. Assays were performed per triplicate.
