# Supplementary material for: The Interaction of RecA With Both CheA and CheW Is Required for Chemotaxis
Source: Front Microbiol. 2020 Apr 7;11:583. doi: 10.3389/fmicb.2020.00583 (PMC7154110; doi:10.3389/fmicb.2020.00583)
Supplement: Supplementary file 8 [file Table_1.pdf]

Supplementary Material

Supplementary Table 1. Bacterial strains and plasmids used in this work.

Strain or plasmid	Relevant characteristic(s)	Source or reference
**Strain**		
**DH5α**	*E. coli supE4 ΔlacU169 (φ80 ΔlacZ ΔM15) hsdR17, recA1, endA1, gyrA96, thi-1, relA1*	Clontech
**ATCC 14028**	*S. enterica* Typhimurium wild type strain	ATCC
**UA1927**	*S.* Typhimurium *recAΩcat*, Cm^R	(Mayola et al., 2014)
**UA1941**	*S.* Typhimurium Δ*recA* Δ*cheA*	This work
**UA1915**	*S.* Typhimurium Δ*rec*A Δ*cheW*	(Mayola et al., 2014)
**UA1942**	*S.* Typhimurium *cheA*::*SNAP tar*::*CLIP*	This work
**UA1943**	*S.* Typhimurium *cheA*::*SNAP recA*::*CLIP*	This work
**UA1944**	*S.* Typhimurium *cheA*::*SNAP tar*::*CLIP* pUA1108	This work
**UA1945**	*S.* Typhimurium Δ*recA cheA*::*SNAP tar*::*CLIP* pUA1108	This work
**UA1946**	*S.* Typhimurium *cheA*::*SNAP tar*::*CLIP* pUA1108 *recA*	This work
**UA1947**	*S.* Typhimurium Δ*recA cheA*::*SNAP tar*::*CLIP* pUA1108 *recA*	This work
**UA1948**	*S.* Typhimurium *cheA*::*SNAP tar*::*CLIP* pUA1108 $recA^{A214V}$	This work
**UA1949**	*S.* Typhimurium Δ*recA cheA*::*SNAP tar*::*CLIP* pUA1108 $recA^{A214V}$	This work
**UA1950**	*S.* Typhimurium *cheA*::*SNAP tar*::*CLIP* pUA1108 $recA^{R222A}$	This work
**UA1951**	*S.* Typhimurium Δ*recA cheA*::*SNAP tar*::*CLIP* pUA1108 $recA^{R222A}$	This work
**UA1952**	*S.* Typhimurium Δ*recA* Δ*cheA* Δ*cheW* pUA1108 *recA::CLIP*	This work
**Plasmid**		
**pKOBEGA**	Vector containing the λ Red recombinase system, Amp^R, temperature sensitive	Generous gift of Prof. G. M. Ghigo (Chaveroche et al., 2000)
**pCP20**	Vector carrying FLP system, $OriV^{ts}$, Amp^R	(Datsenko and Wanner, 2000)
**pKD4**	Vector carrying FRT-Kan construction, Amp^R, Kan^R	(Datsenko and Wanner, 2000)
**pUA1108**	pGEX 4T-1 derivative plasmid carrying only the P*tac* IPTG-inducible promoter and the $lacI^q$ gene; used as overexpression vector, Amp^R	(Mayola et al., 2014)
**pGEMT**	Cloning vector, Amp^R	Promega
**pUA1135**	pGEMT derivative plasmid containing the *SNAP*-tag gene and kanamycin cassette flanked with FRT sequences under the control of the P*tac* promoter, $Amp^R Kan^R$	This work
**pUA1136**	pGEMT derivative plasmid containing the *CLIP*-tag gene and kanamycin cassette flanked with FRT sequences under the control of the P*tac* promoter, $Amp^R Kan^R$	This work
**pUA1130**	pUA1108 derivative plasmid containing the native *S.* Typhimurium *recA* gene under the control of the P*tac* promoter, Amp^R	(Mayola et al., 2014)
**pUA1137**	pUA1108 derivative plasmid containing the *S.* Typhimurium $recA^{A214V}$ mutant under the control of the P*tac* promoter, Amp^R [pUA1108 $recA^{A214V}$]	This work
**pUA1138**	pUA1108 derivative plasmid containing the *S.* Typhimurium $recA^{R222A}$ mutant under the control of the P*tac* promoter, Amp^R [pUA1108 $recA^{R222A}$]	This work
**pUA1139**	pUA1108 derivative plasmid containing the *S.* Typhimurium $recA^{R176A}$ mutant under the control of the P*tac* promoter, Amp^R [pUA1108 $recA^{R176A}$]	(Irazoki et al., 2016)
**pUA1140**	pUA1108 derivative plasmid containing the *S.* Typhimurium *recA::CLIP* under the control of the P*tac* promoter, Amp^R	This work

Chaveroche, M. K., Ghigo, J. M., and d'Enfert, C. (2000). A rapid method for efficient gene replacement in the filamentous fungus *Aspergillus nidulans*. *Nucleic Acids Res.* 28, E97. doi:10.1093/nar/28.22.e97

Datsenko, K., and Wanner, B. L. (2000). One-step inactivation of chromosomal genes in *Escherichia coli* K-12 using PCR products. *Proc. Natl. Acad. Sci. U. S. A.* 97, 6640–6645. doi:10.1073/pnas.120163297.

Irazoki, O., Aranda, J., Zimmermann, T., Campoy, S., and Barbé, J. (2016). Molecular interaction and cellular location of RecA and CheW proteins in *Salmonella enterica* during SOS response and their implication in swarming. *Front. Microbiol.* 7, 1560. doi:10.3389/fmicb.2016.01560.

Mayola, A., Irazoki, O., Martínez, I. A., Petrov, D., Menolascina, F., Stocker, R., et al. (2014). RecA protein plays a role in the chemotactic response and chemoreceptor clustering of *Salmonella enterica*. *PLoS One* 9, e105578. doi:10.1371/journal.pone.0105578.
