# Supplementary material for: The Interaction of RecA With Both CheA and CheW Is Required for Chemotaxis
Source: Front Microbiol. 2020 Apr 7;11:583. doi: 10.3389/fmicb.2020.00583 (PMC7154110; doi:10.3389/fmicb.2020.00583)
Supplement: Supplementary file 9 [file Table_2.pdf]

Supplementary Material

Supplementary Table 2. Primers used in this work.

Primer name	Sequence	Application
Tar_clip_F	agtgaacgctcagtccggcaatacgccgcagtcattagccgccagggatgatgcgaactgggaaaccttcGCCTCCGCCGCCGCCTCCATGGACAAAGAC	Oligonucleotides to introduce CLIP-tag after *tar* gene
Tar_clip_R2	tttttgcttttatctatgcgaaccagacgaaaggtatcggcgggttcgcaaattaatcgataaccgacagcgcacgtcgaATGGGAATTAGCCATGGTCC	
R_clip_F	taatcagaatgccacgcccgatttcgccgttgacgatagcgaaggcgttgcagaaaccaacgaagattttGCCTCCGCCGCCGCCTCCATGGACAAAGAC	Oligonucleotides to introduce CLIP-tag after *recA* gene
R_clip_P2	cataaatgcagcccttgatggtaatttaacgttttgctgaatggcggcttcgttttgcccgccccaccatcacctgatgaATGGGAATTAGCCATGGTCC	
W_snap_F	ggtgaatatcgaaaaactgcttaacagcgaagagatggcgctgctggatatcgcagcatcacacgtcgcggcctccgccgccgcctccatggacaaagac	Oligonucleotides to introduce SNAP-tag after *cheW* gene
W_snap_P2	cgatgaagaggcactctcaccgctggcggaagcataacggtgaatattgccggatggcgcgacgccatccggcaacgttaATGGGAATTAGCCATGGTCC	
A_snap_F	cgcgctgattgttgatgtttccgcattgcagggtttaaaccgcgaacaacgtatggcgatcacagccgccgcctccgccgccgcctccatggacaaagac	Oligonucleotides to introduce SNAP-tag after *cheA* gene
A_snap_P22	caggaattcctgacctgacggctcgccggccagtttgcttacattactcataccggtcatattattcccttctcactcaaATGGGAATTAGCCATGGTCC	
cApKO3BF	agggatccTTCGATAATTCACCAGAATCAG	Oligonucleotides for checking 500 bp downstream and upstream of its STOP codon
cApKO3BR	agggatccGATGTGGTGAAACGTAACAT	
cheA_fw	acacaggaaacagtacatATGAGCATGGATATTAGCG	Oligonucleotid to amplify since start of *cheA*
cWpKO3BF	agggatccCATTCTGGAGTAAATCCGT	Oligonucleotides for checking 500 bp downstream and upstream of its STOP codon
cWpKO3BR	agggatccACCGGTATGAGTAATGTAAGC	
pNAS_RecAf	ttcacacaggaaacagtacaATGGCTATCGACGAAAAC	Oligonucleotides for checking *recA* gene from its start and final *recX* gene
recX_HF_f	gggatcgcggccgcggaccggatccTCAATCTGCAAAATTTCGCC	
tarpKO3BF	AGggatccATGGCGATACTGTAAGGTTCT	Oligonucleotides for checking 500 bp downstream and upstream of its STOP codon
tarpKO3BR	agggatccACTAACATTCTGGCGCTGA	
M304A F	TACCCAGTCAgcgTTGGCCCAGC	Oligonucleotides for CheA M303A mutant derivative construction
M304A R	ATCACTAACTCGCCGACC	
L312A F	TTCTAACGAGgcgGACCCGGTAAACC	Oligonucleotides for CheA L311A mutant derivative construction
L312A R	CGCTGGGCCAACATTGAC	
G538A F	TCGCGTGGCGgcgGAAGTTTTTA	Oligonucleotides for CheA G537A mutant derivative construction
G538A R	ACCGACATCCCATCGAGG	
D588A F	GTTTGACGTGgcgGGGGCGAAAAC	Oligonucleotides for CheA D587A mutant derivative construction
D588A R	ACTTTCCACAATTCGACC	
K591A F	GGACGGGGCGgcaACCGAAGCCA	Oligonucleotides for CheA K590A mutant derivative construction
K591A R	ACGTCAAACACTTTCCACAATTCGAC	

T592A F	CGGGGCGAAAgccGAAGCCACGC	Oligonucleotides for CheA T591A mutant derivative construction
T592A R	TCCACGTCAAACACTTTCCACAATTCGACC	
S629A F	AAATCTGGAAgccAATTATCGCAAGGTACCGG	Oligonucleotides for CheA S628A mutant derivative construction
S629A R	TTGACCACCACCTGGTGC	
S647A F	GGGCGACGGCgccGTCGCGCTGA	Oligonucleotides for CheA S646A mutant derivative construction
S647A R	AGGATCGTCGCGGCGGAAATC	
F204A_F	TGGCGTGATGgcgGGTAACCCGG	Oligonucleotides for RecA F203A mutant derivative construction
F204A_R	ATCTTCATACGGATCTGG	
I229A_F	TATCCGTCGTgcgGGCGCGGTGA	Oligonucleotides for RecA I228A mutant derivative construction
I229A_R	TCAAGACGAACAGAGGCG	
R244A_F	TAGCGAAACGgcgGTGAAAGTGGTGAAAAACAA AATC	Oligonucleotides for RecA R243A mutant derivative construction
R244A_R	CCCACGACATTATCGCCC	
F256A_F	CGCCGCGCCGgcgAAGCAGGCCG	Oligonucleotides for RecA F255A mutant derivative construction
F256A_R	ATTTTGTTTTTCACCACTTTCACACGCGTTTC	
Q258A_F	GCCGTTTAAGgcgGCCGAGTTCC	Oligonucleotides for RecA Q257A mutant derivative construction
Q258A_R	GCGGCGATTTTGTTTTTC	
K287A_F	GCTGATCGAGgcgGCGGGCGCAT	Oligonucleotides for RecA K286A mutant derivative construction
K287A_R	TTCTCTTTCACGCCCAGG	
Q301A_F	GAAGATTGGCgcgGGTAAAGCGAACG	Oligonucleotides for RecA Q300A mutant derivative construction
Q301A_R	TCGCCGTTGTAGCTGTAC	
